# Early Everolimus Initiation Fails to Counteract the Cytotoxic Response Mediated by CD8^+^ T and NK Cells in Heart Transplant Patients

**DOI:** 10.3389/fimmu.2018.02181

**Published:** 2018-09-26

**Authors:** Beatriz Díaz-Molina, Paula Diaz-Bulnes, Reyes Carvajal Palao, Maria José Bernardo, Ramón M. Rodriguez, Viviana Corte-Iglesias, Cesar Moris de la Tassa, Jose Luis Lambert, Beatriz Suarez-Alvarez

**Affiliations:** ^1^Advanced Heart Failure and Transplant Service, Department of Cardiology, Hospital Universitario Central de Asturias, Oviedo, Spain; ^2^Translational Immunology Laboratory, Instituto de Investigación Sanitaria del Principado de Asturias (ISPA), Hospital Universitario Central de Asturias, Oviedo, Spain; ^3^Department of Cardiology, Hospital Universitario Central de Asturias, Oviedo, Spain; ^4^Faculty of Health Sciences, Universidad Católica San Antonio de Murcia, Murcia, Spain

**Keywords:** everolimus, heart transplantation, cytotoxicity, DNA methylation, CD8^+^ T cells, NK cells, mTOR inhibitors

## Abstract

The positive long-term effects of conversion to everolimus (EVL) after heart transplantation (HT) have been evaluated in several studies. However, the timing of EVL initiation, the best way to combine it with other immunosuppressive treatments, and the impact of these combinations on the immune response are poorly understood aspects. Here, we analyzed the immune phenotype and function of HT patients (*n* = 56) at short and long terms (prospective and retrospective cohorts), taking into account the time of EVL initiation: early (3 months post-transplant, EVL-E group) or late (>1 year post-transplant, EVL-L group) compared with mycophenolate mofetil treatment (MMF group). We show that early EVL conversion from MMF allows the increase of cytotoxic (CD56^dim^ CD16^+^) NK and effector-memory (EM, CD45RA^−^ CCR7^−^) CD8^+^ T cell subsets, which show a significantly higher level of expression of cytotoxic molecules, IFN-γ production and degranulation ability under activation. NK cell expansion is accompanied by an altered balance of receptor expression, increasing the activation state, and lytic activity of those cells. Those changes are detected after as little as 1 month after EVL conversion in association with the expansion of regulatory T cells and the decrease in B cell frequency. However, no changes in the immune cells subsets were observed after late EVL initiation (EVL-L) compared with the MMF group. Our results imply that only early EVL conversion induces key changes in the post-transplant immune response, preserving an efficient anti-viral response, but simultaneously showing a limited ability to counteract the cytotoxic response to the allograft.

## Introduction

Current immunosuppressive regimens after heart transplantation (HT) had allowed acceptable one-year survival rates (around 85%), but complications such as cardiac allograft vasculopathy (CAV), cytomegalovirus infection (CMV) and calcineurin-inhibitor (CNI)-induced nephrotoxicity continue to be serious obstacles to the long-term survival of these patients (1).

Treatment with everolimus (EVL), a mammalian target of rapamycin (mTOR) inhibitor, has shown itself to be effective in reducing CAV progression and CMV infection, and in decreasing CNI-induced nephrotoxicity in combination with mycophenolate mofetil (MMF) (2–5). Initial studies showed that EVL in combination with cyclosporine and corticosteroids was more effective at reducing the severity of CAV than azathioprine, but not at improving renal function (6). Subsequent studies showed that, in combination with MMF, EVL can ameliorate CNI-induced nephrotoxicity although there is a high risk of acute cellular rejection (ACR) (7). One point of controversy is the timing of introduction of EVL, which can affect its short- and long-term effects. EVL conversion from MMF during the maintenance period is mainly employed to avoid the progression of CAV and the loss of renal function, or in cases of repeated ACR (8). However, it has been suggested that the effects of this drug might be more pronounced if its administration were initiated during the first months post-transplant. In fact, it has been proposed that all *de novo* HT patients could be candidates for EVL initiation, except those with baseline proteinuria or uncontrolled severe hyperlipidemia (9). A recent study showed that EVL initiation 4–6 weeks after HT with reduced-dose CsA led to better anti-rejection efficacy and a better safety profile, although CMV infection is more common than *de novo* EVL initiation (10). However, there is contradictory evidence about this, which leaves many questions unanswered, and explains why no clear strategy has yet emerged (11). In fact, data from the ISHL registry show that only about 13% of HT patients receive mTOR inhibitors as part of their long-term immunosuppressive maintenance regimen, including those receiving it as salvage therapy due to the development of CAV or renal insufficiency (12).

It is widely accepted that the mTOR signaling pathway is crucial for the modulation of the innate and adaptive immune systems (13–15). mTOR is a ubiquitously expressed serine/threonine-protein kinase whose downstream signaling regulates diverse processes such as cell metabolism, proliferation, migration, protein translation, and survival in response to various environmental stimuli (e.g., availability of nutrients, growth factors, cytokines, and antigen-receptor signaling). Activation of the mTOR pathway is essential for maturation, development and cytokine production by dendritic cells (16–18). In T lymphocytes, mTOR directs the polarization of CD4^+^ T cells toward Th1, Th2, Th17, and regulatory T cells (Tregs) (19). In fact, mTOR-deficient CD4^+^ T cells fail to differentiate into helper T cells even in the presence of cytokines (20). Moreover, the expansion and migration of CD8^+^ T cells into inflammatory tissues, and their differentiation into effector and memory CD8^+^ T cells are also regulated by mTOR (21–24). One of the best-analyzed effects of mTOR inhibitors on the immune system is the expansion of Tregs arising from the immunosuppressive ability of these cells (25, 26). In response to IL-2, the level of activation of the PI3K/Akt/mTOR pathway in Tregs is lower than in conventional T cells, making these Tregs more insensitive to the anti-proliferative effects of m-TOR inhibitors, thereby inducing their expansion. Additionally, mTOR inhibitors also enhance the expression of the FoxP3 transcription factor (27).

For this study, we hypothesized that the timing of EVL initiation after HT could modify the immune phenotype in a variety of ways and thereby the post-transplant immune response conditioning the clinical outcomes. We focus on the cytotoxic immune response mediated by CD8^+^ T and NK cells, which play an essential role in long-term cellular and antibody-mediated rejection, respectively, and in the anti-viral immune response. To address these matters, we analyzed the immune function of HT patients with respect to the timing of EVL initiation and the outcomes during the first months post-transplant and over the long term.

## Materials and methods

### Study populations and design

HT patients were enrolled from the Heart Transplant Unit, Hospital Universitario Central de Asturias, Oviedo, Spain. This study was carried out in accordance with the recommendations of the European Community Guidance on Good Clinical Practice^1^. The protocol was approved by the local ethical committee. All subjects gave written informed consent in accordance with the Declaration of Helsinki^2^.

**Figure 1:**
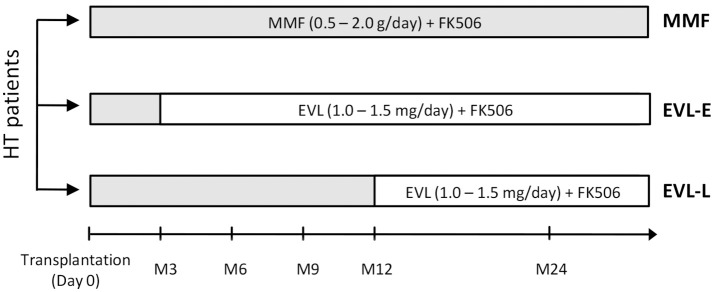
Design of the retrospective study. HT (heart transplant) patients enrolled with respect to the immunosuppressant treatment and the timing of EVL initiation. MMF group (*n* = 20), patients under MMF treatment; EVL-E group (*n* = 12), patients converted to EVL 3 months after HT; EVL-L (*n* = 8), patients converted to EVL >1 year after HT. MMF, mycophenolate mofetil; EVL, everolimus.

The retrospective study was designed to evaluate the long-term effects of EVL versus MMF treatment in relation to the time of EVL initiation. All HT participants (*n* = 40) fulfilled the following inclusion criteria: (1) adult transplanted between 2008 and 2012 with no other associated transplants, tumors, or infections currently being treated; (2) with more than 2 years post-HT at the time the sample was collected; and (3) free from acute rejection in the 3 months before the analysis. All patients received induction therapy with basiliximab and prophylactic treatment of CMV infection in accordance with the center's standard practice. Steroid treatments were gradually reduced during the first year post-transplant until complete withdrawal was achieved. In the first 3 months after transplant, all patients received standard triple immunosuppressive therapy with MMF, FK-506, and steroids. After that, patients were randomized to be assigned to receive maintenance MMF therapy (0.5–2.0 g/day, MMF group, *n* = 20) or to be initiated in an early conversion to EVL (1.0–1.5 mg/day, EVL-E group, *n* = 12) (Figure 1). A third group of patients converted to EVL from MMF during the maintenance period (at least 1 year post-transplant) was also recruited to analyze the effects of late EVL conversion (EVL-L group, *n* = 8) (Figure 1). Late conversion to EVL from MMF was done to prevent potentially adverse conditions in patients with a decline in renal function or with incipient CAV development. In all patients, MMF or EVL was administered with low doses of tacrolimus (FK-506, 1.0–5.0 mg/day). Blood samples were collected on a single occasion, 2 years post-transplant and at least 1 year after EVL initiation in the EVL-L group. Clinical parameters were measured at the time of sample collection and all patients were followed up for at least another two years (Table 1). Acute cellular rejection was monitored by endomyocardial biopsies, and CAV was checked by coronary angiography.

**Table 1:** Clinical characteristics of HT patients, by treatment group.

	**MMF (*n* = 20)**	**EVL-E (*n* = 12)**	**EVL-L (*n* = 8)**
Donor age; median (IQ), y	47.5 (22–58)	44 (14–59)	46.5 (24–59)
Recipient age; median (IQ), y	54 (38–69)	57 (44–69)	62.5 (51–64)
Donor gender; n (M:F)	11:9	10:2	5:3
Recipient gender; n (M:F)	14:6	11:1	6:2
HLA mismatches; median (IQ)	4.5 (2–6)	6 (4–6)^*^	5 (4–6)
Heart disease (etiology); *n* (%)			
Dilated cardiomyopathy	16 (80)	4 (33)^*^	2 (25)^†^
Ischemic cardiomyopathy	3 (15)	6 (50)^*^	4 (50)
Others	1 (5)	2 (16.6)	2 (25)
Medical history; *n* (%)			
Hypertension	8 (40)	5 (41.6)	4 (50)
Diabetes	6 (30)	2 (16.6)	2 (25)
Dyslipidemia	5 (25)	4 (33.3)	3 (25)
Smoker	9 (45)	1 (8.3)^*^	3 (37.5)
Medications; *n* (%)			
Corticosteroids	4 (20)	1 (8.3)	1 (12.5)
β-blocker	2 (10)	0	0
ACE inhibitor	6 (30)	4 (25)	1 (12.5)
ARAII	3 (15)	0 (15)	3 (37.5)^#^
Statin therapy	14 (70)	9 (75)	5 (62.5)
Biochemical data; median (IQ)			
Total cholesterol; mg/dL	155.5 (105–276)	158.5 (114–280)	182 (170–273)^†^
HDL; mg/dL	50 (33–85)	44.5 (21–83)	47 (35–66)
LDL; mg/dL	89 (53–184)	70 (48–186)	110 (88–175)^†^
Triglycerides; mg/dL	102 (38–275)	139.5 (68–351)	163 (78–288)
Serum creatinine; mg/dL	1.19 (0.39–2.97)	1.17 (0.91–1.7)	1.57 (1.05–2.86)
Immunosuppressant blood levels; median (IQ)			
FK506 level; ng/ml	6.80 (3.3–14.4)	8.3 (4.2–10.6)	7.05 (4.2–11.8)
Everolimus level; ng/ml	0	3.38 (1.93–4.75)	3.85 (2.7–6.42)
Post-transplant follow-up; median (IQ), y	6.95 (5.7–9.3)	4.8 (3.8–5.5)^*^	6.25 (5.7–7.8)^#^
Rejection; *n* (%)			
Acute cellular rejection (≥2R, %)	7 (35)	3 (25)	0
CAV	2 (10)	2 (16.6)	1 (12.5)
CMV infection; n (%)	5 (25)	0	1 (12.5)
Tumors; n (%)	3 (15)	2 (16.6)	1 (12.5)

*MMF, mycophenolatemofetil; EVL, everolimus; CAV, chronic allograft vasculopathy; CMV, cytomegalovirus; n.d., not determined*.

* *p < 0.05 between MMF group and early EVL group*;

# *p < 0.05 between early EVL group and late EVL group*;

† *p < 0.05 between MMF group and late EVL group*.

A second prospective study was carried out with 16 HT patients between January 2012 and December 2013 in order to determine whether any long-term effect after early EVL conversion could also be detected during the first year post-transplant. All patients received induction therapy with basiliximab and initial triple therapy with MMF, FK506 and steroids. Three months after transplantation, all patients were converted to EVL from MMF and blood samples were taken on four occasions: before EVL initiation (Pre-E), and 1, 3, and 9 months after EVL conversion (Post-E1, Post-E2, Post-E3, respectively). Baseline characteristics are summarized in Table S1.

### Peripheral blood immunophenotyping

Flow cytometry immunophenotyping was carried out on whole-blood samples using specific monoclonal antibodies (mAbs, Table S2). 100 μL of EDTA-treated peripheral blood was stained with mAbs for 30 min at 4°C. Red blood cells were lysed with FACS lysing solution (BD Biosciences, CA, USA) and analyzed in the FACSAria II cytometer using FACSDiva™ software (BD Biosciences). Isotype controls were used to define marker settings. The gating strategy used to identify the subtypes of T cells by flow cytometry was as follows: PBMCs were selected according to physical parameters (forward and side scatter) and death cells were rejected by positive 7-AAD staining (Biolegend, San Diego, CA, USA). CD4^+^ and CD8^+^ T lymphocytes were selected according to the combined expression of CD3 and CD4 or CD8 markers (Figure S1A). Further, gated CD4^+^ and CD8^+^ T cells were stained using CCR7 (C-C motif chemokine receptor-7) and CD45RA surface markers. These markers have been reported to classify T cells in four subsets with diverse functional properties; naive (N: CD45RA^+^ CCR7^+^), central memory (CM, CD45RA^−^ CCR7^+^), effector memory (EM, CD45RA^−^ CCR7^−^) and terminally differentiated effector memory cells re-expressing CD45RA (TEMRA, CD45RA^+^ CCR7^−^) (28). Subtypes of NK cells were determined according to the combined expression of CD56 and CD16 markers, after previous gated of lived and CD3^−^ cells (Figure S1B). Three different NK cells subsets were analyzed; highly cytotoxic (CD56^dim^ CD16^+^), mainly producing cytokines (CD56^bright^ CD16^+/−^) or associated with chronic viral infections (CD56^−^ CD16^+^). For the analysis of Tregs, we used the CD3^+^, CD4^+^, CD25^high^, and CD127^low/−^ markers. CD4^+^ T cells were gated using the combined expression of CD3^+^ and CD4^+^ markers and, from this subset, cells expressing high levels of CD25 and low or absent CD127 expression were identified (Figure S1C). The FoxP3 transcription factor expression in those cells was corroborated in some samples but it was not used to identify Tregs due to its expression is dependent of the inflammatory microenvironment. Moreover, some effector (non-suppressive) activated T cells also express that transcription factor. The lack or low expression of CD127 (IL-7R) marker in CD25^high^ T cells identify human Tregs with a potent suppressive function (29) and a good correlation between peripheral CD4^+^ FoxP3^+^ Tregs and CD4^+^ CD25^high^ CD127^low/−^ Tregs has been observed in health donors confirming the use of those markers (30).

Determination of the absolute counts of immune cells subsets in peripheral blood was determined using BD Trucount™ Tubes and BD Multiset™ (BD Biosciences) analysis. To this end, 50 μl of EDTA-treated peripheral blood was stained with the following mAb cocktails: MultiTEST CD3-FITC (clone SK7)/CD8-PE (clone SK1)/CD45-PERCP (clone 2D1)/CD4-APC (clone SK3) and MultiTEST CD3-FITC (clone SK7)/CD16+56-PE (clone B73.1+NCAM16.2)/CD45-PERCP (clone 2D1) / CD19-APC (clone SJ25C1) (BD Biosciences). The absolute number (cells/μl) of positive cells in the sample was calculated by comparing cellular events to known beads events.

### CD107a-degranulation assays, IFN-γ production and cytotoxic molecule staining

For *in vitro* assays, peripheral blood mononuclear cells (PBMCs) were isolated by Ficoll (Lymphoprep) density-gradient centrifugation and incubated on plates coated with anti-CD3 (3 μg/ml) and anti-CD28 (1 μg/ml) mAbs (Biolegend) or with IL-2 (100 U/ml) and IL-15 (10 ng/ml)(PeproTech EC Ltd, London, UK) for activation of CD8^+^ T and NK cells, respectively. CD107a-FITC (clone H4A3) antibody was added to the wells followed by monesin (6 μg/ml, Biolegend) and cultured for 6 h before analysis on a Gallios Flow Cytometer using Kaluza 1.3 analysis software (Beckman Coulter, CA, USA). For IFN-γ production, activated CD8^+^ T lymphocytes were treated for 5 h with brefeldin A (10 μg/ml, Biolegend), and cell surfaces were stained with CD8-PE and CD3-PerCP mAbs, followed by intracellular staining with anti-INFγ-FITC for 30 min at 4°C. Similarly, PBMCs were incubated with CD8-PE, CD3-PerCP and CD16-APC mAbs, followed by intracellular staining with granzyme B-PE and perforin-FITC mAbs (Biolegend). In both cases, a fixation / permeabilization kit (Immunostep, Salamanca, Spain) was used and the 7-Aminoactinomycin D (7AAD) fluoresecent marker (Biolegend) was included to excluded dead cells from the analysis. Cells were further analyzed by flow cytometry.

### CFSE-based proliferation assay

PBMCs were isolated from six healthy donors from the Asturias Transfusion Centre, Oviedo, Spain, after obtaining their informed consent. CD4^+^ and CD8^+^ T cells were isolated using human CD4 and CD8 microbeads (Myltenyi Biotec, Bergisch Gladbach, Germany), respectively, and in all cases purity was >95%. After that, cells were labeled with CFSE (1.25 μM, Biolegend). 10 × 10^5^ CFSE-labeled cells were incubated overnight on plates previously coated with anti-CD3 and anti-CD28 mAbs in the absence or presence of EVL, FK-506, and MMF for 5 days, washed and stained with CD4-PE, CD3-PerCP, and CD8-APC (Biolegend) for further analysis by flow cytometry. After surface staining, cells were stained with 7-AAD marker (Biolegend) in PBS for 30 min in order to exclude death cells (7-AAD positive cells).

### Pyrosequencing

Genomic DNA was extracted from PBMCs using a *DNeasy*® Blood & Tissue Kit (Qiagen, Valencia, CA, USA). Purified DNA (1 μg) was modified using a DNA Methylation-Gold™ kit (Zymo Research, CA, USA) and further amplified with specific biotinylated primers (details available upon request) designed by PyroMark Assay Design Software 2.0 (Qiagen, CA, USA). Pyrosequencing was performed with the PyroMark™ Q24 System version 2.0.6, and analyzed with PyroMark Q24 2.0 software (Qiagen).

### Statistical analysis

Data are expressed as the mean and standard deviation for normally distributed continuous variables, medians, and interquartile ranges for non-normally distributed continuous variables, and frequencies (percentages) for categorical variables. Differences between groups were assessed using the appropriate test with values of *p* < 0.05 being considered to indicate statistical significance. Analyses were carried out with SPSS v17.0 (SPSS Inc., Chicago, IL, USA).

## Results

### Effects of EVL conversion on the immune phenotype

**Figure 2:**
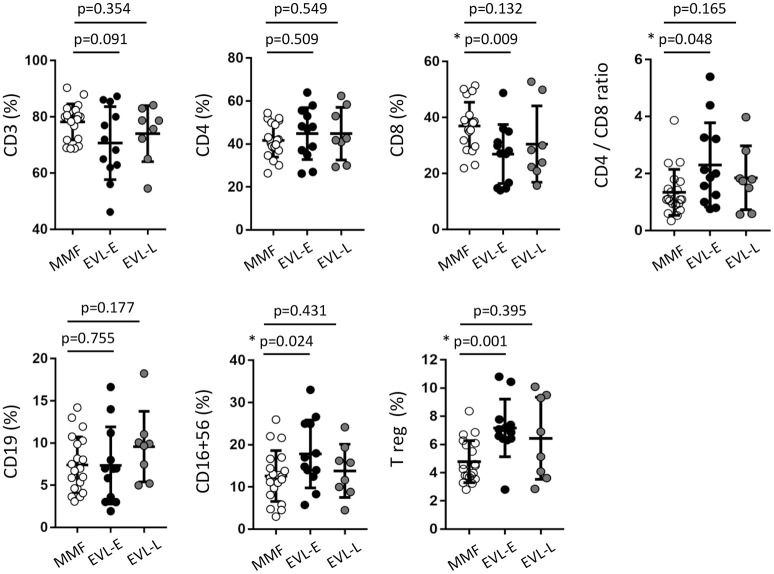
Immune phenotype in peripheral blood of HT patients, by treatment group. The percentage of the immune cell subsets was determined in 50 μl of peripheral blood obtained from HT patients at >1 year post-transplant who were receiving therapy with MMF (MMF group, *n* = 20) or who were converted to EVL early (EVL-E, *n* = 12) or late (EVL-L, *n* = 8). Each circle represents one HT patient, and the mean and standard deviation are depicted as black bars. Significant differences between groups were determined by the Mann–Whitney *U*-test. *p < 0.05.

Our main aims were to determine whether EVL conversion from MMF induces long-term changes in the immune subsets and whether such changes are dependent on the timing of EVL initiation. For this purpose, we analyzed the immunophenotype in peripheral blood of patients from the three previously described groups (Figure 1). No significant differences were observed between HT patients under MMF treatment (MMF group) and those converted to EVL during the maintenance period (EVL-L group) (Figure 2). However, patients converted to EVL early (EVL-E group) showed a significant reduction in the percentage and absolute number of CD8^+^ T cells (*p* = 0.009), while the proportion of CD4^+^ T cells remained at similar levels, as shown by the highest CD4^+^/CD8^+^ ratio observed in the EVL-E group (Figure 2 and Figure S2). Moreover, the percentages of NK cells (CD16^+^ CD56^+^, *p* = 0.024) and regulatory T cells (Treg, *p* = 0.001) also increased significantly after early EVL conversion. No changes in the percentages of CD3^+^ cells (pan-T cells) and CD19^+^ cells (pan-B cells) were detected, but the absolute number of CD3^+^ T cells was significantly decreased (*p* = 0.003) mainly due to the reduced abundance of CD8^+^ T cells (Figure S2).

**Figure 3:**
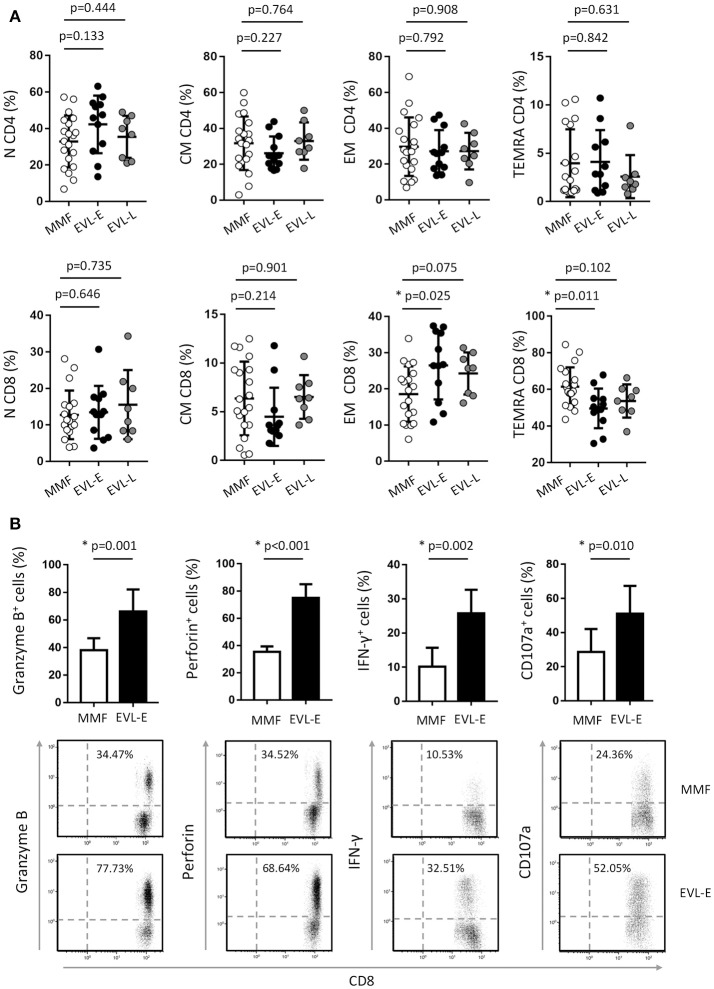
Effect of EVL on the cell subsets of CD4^+^ and CD8^+^ T cells and functional properties of cytotoxic CD8^+^ T cells. **(A)** Distribution of CD4^+^ and CD8^+^ T lymphocytes into naive (N, CD45RA^+^ CCR7^+^), central memory (CM, CD45RA^−^ CCR7^+^), effector-memory (EM, CD45RA^−^ CCR7^−^) and terminally differentiated effector-memory re-expressing CD45RA (TEMRA, CD45RA^+^ CCR7^−^) subsets, in HT patients from MMF (*n* = 20), EVL-E (*n* = 12) and EVL-L (*n* = 8) groups. **(B)** Histograms represent the percentage of CD8^+^ T lymphocytes expressing cytolytic molecules (Granzyme B and Perforin), producing IFN-γ cytokine or with the ability to degranulate (show by the cell-surface expression of the CD107a marker) for each patient from the MMF (*n* = 20) and EVL-E (*n* = 12) groups. Dot-plots are representative of an independent experiment from each treatment group. *p < 0.05.

To examine in greater detail the changes in the T cell subtypes after EVL initiation, we analyzed the phenotypic characteristics of CD4^+^ and CD8^+^ T cells with respect to the CCR7 (C-C motif chemokine receptor-7) and CD45RA surface markers. Early conversion to EVL is associated with wide variations in CD8^+^ T cell subtypes, whilst all CD4^+^ T cell subsets were unchanged (Figure 3A). HT patients who were converted to EVL at 3 months post-transplant (EVL-E group) showed a significant increase in the percentages of effector-memory (EM) cells (*p* = 0.025) and a decrease in those of the terminally differentiated effector-memory cells re-expressing CD45RA (TEMRA; *p* = 0.011) in the CD8^+^ T cell compartment (Figure 3A). This trend was also observed in patients who initiated EVL during the maintenance period (EVL-L group) but was less pronounced and not statistically significant (Figure 3A).

Considering the groups described, no significant differences in the clinical outcomes (ACR, CAV, or CMV infection) were observed. However, more patients in the MMF group had CMV infection than did those in the EVL-E group, in which no patient showed an infection. We also observed that, after late EVL conversion (EVL-L), patients had higher levels of total cholesterol, LDL, triglycerides and serum creatinine, although a higher incidence of CAV was not detected. Significant differences in the post-transplant follow-up time were due to the protocol of EVL initiation 3 months post-HT being more recently introduced in our hospital (Table 1).

### Limited efficiency of EVL in inhibiting cytolytic properties of CD8^+^ T cells

Effector CD8^+^ T cells are major players in allograft rejection and an increase in the number of memory CD8^+^ T cells is associated with long-term allograft dysfunction (31). We questioned whether the increase of EM CD8^+^ T cells observed after early EVL conversion is due to a weaker inhibition of the functionality and proliferation of these cells by EVL compared than that in patients receiving MMF treatment. First, we analyzed the expression of effector molecules (granzyme and perforin) and the IFN-γ pro-inflammatory cytokine in activated CD8^+^ T cells of patients from both groups. We observed that HT patients who converted early from MMF to EVL (EVL-E group) showed a significant increase of cells expressing cytotoxic molecules (granzyme B, *p* = 0.001; perforin, *p* < 0.001) and greater frequency of cells producing the pro-inflammatory cytokine IFN-γ (*p* = 0.002, Figure 3B). Moreover, these results are associated with enhanced degranulation by CD8^+^ T cells (*p* = 0.010), as shown by the CD107a expression levels (32) (Figure 3B).

We analyzed the *in vitro* effect of EVL on the proliferation of CD4^+^ and CD8^+^ T cells compared with MMF or FK506 treatment. EVL more efficiently blocked the cell proliferation of CD4^+^ than CD8^+^ T cells, whilst FK-506 had the opposite effect (Figure S3). MMF, at doses as low as 1 μM, was the most potent drug for counteracting the proliferation of both T cell types. Therefore, we demonstrate that EVL has a more limited ability to inhibit the cell proliferation of CD8^+^ T lymphocytes than the other immunosuppressive treatments. In other words, the increase in the percentage of EM CD8^+^ T cells in EVL-E group patients could be due to the lesser ability of this drug to reduce their proliferation under activation.

### Phenotypic and functional characteristics of NK cells are altered after early EVL treatment

**Figure 4:**
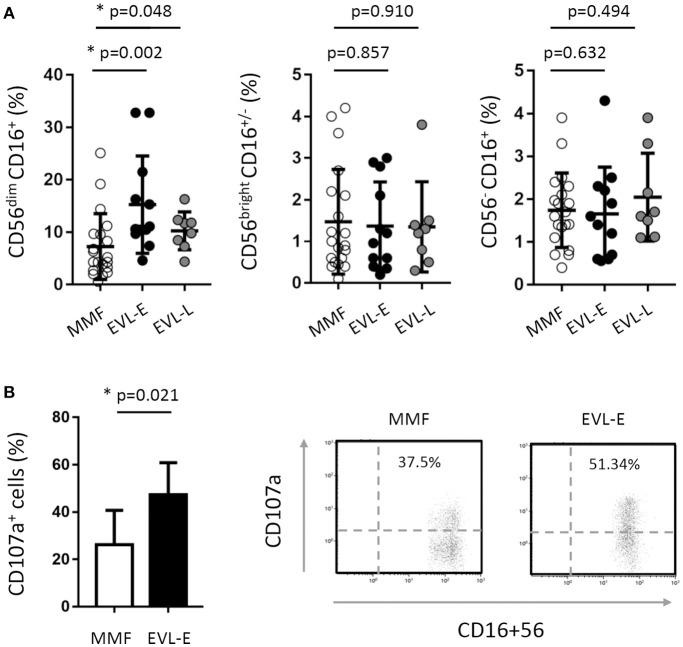
Effect of EVL on the subsets and functionality of NK cells. **(A)** Distribution of NK cell subsets into CD56^dim^ CD16^+^ (cytotoxic cells), CD56^bright^ CD16^+/−^ (cells producing cytokines) and CD56^−^ CD16^+^ (cells expanded in chronic viral infections) in HT patients from the MMF (*n* = 20), EVL-E (*n* = 12) and EVL-L (*n* = 8) groups. Each circle represents one HT patient, and the mean and standard deviation are depicted as black bars. Significant differences between groups were determined by the Mann–Whitney *U*-test. *p < 0.05. **(B)** Degranulation (CD107a^+^ cells) of NK cells under activation with IL-2 + IL-15 cytokines for each patient from the MMF (*n* = 20) and EVL-E (*n* = 12) groups. Significant differences between groups were determined by Student's *t*-test or the Mann–Whitney *U*-test. *p < 0.05. Dot-plots show one representative experiment of each group.

As shown above, HT patients who converted early to EVL from MMF (EVL-E group) had an increased percentage of NK cells. Examining this in more detail, we determined the different NK cells subsets according to the expression of the CD16 and CD56 markers. The most cytotoxic subset (CD56^dim^ CD16^+^) was significantly increased (*p* = 0.002) after early EVL conversion relative to the MMF group, whilst NK cells associated with a higher cytokine production (CD56^bright^ CD16^+/−^) or chronic viral infections (CD56^−^ CD16^+^) were present at similar levels in both groups (Figure 4A). As we previously reported, late EVL conversion has no effect on the percentage and phenotype of NK cells compared with patients under MMF treatment (Figure 4A).

The cytolytic activity of NK cells is regulated by a dynamic balance of signals triggered by a variety of activating and inhibitory receptors. We analyzed the cell surface expression of activating (NKG2D, NKp46, NKp30, 2B4, and DNAM) and inhibitory (KIR2DL1/2/3, KIR3DL1, NKG2A) receptors in patients from the EVL-E group compared with MMF-treated patients. The level of expression on the cell surface of inhibitory KIR3DL1 and NKG2A receptors was significantly lower in NK cells from EVL-E group patients than in those from MMF group patients (Table 2). No changes were seen in the expression of the analyzed activating receptors. A decrease in the level of inhibitory receptor expression could tip the balance toward the activating signals favoring the activation and cytotoxicity ability of NK cells. To corroborate whether these phenotypic changes in NK cells are associated with their functional activity we carried out degranulation assays in NK cells isolated from EVL-E and MMF group patients. We found that the NK cells from EVL-E patients had significantly higher levels of CD107a expression (*p* = 0.021) than those from patients treated with MMF (Figure 4B).

**Table 2:** Expression of NK cell receptors in HT patients from the prospective study.

	**MMF**		**EVL-E**	
	**Median**	**IQ range**	**Median**	**IQ range**
CD16	8.36	1.62–11.77	7.96	1.65–10.03
CD158a+b	39.73	23.75–69.48	33.08	18.98–68.91
KIR3DL1	32.59	19.25–51.32	9.18^*^	3.77–15.96
NKG2A	47.6	31.29–76.04	31.59^*^	21.52–36.89
NKG2D	95.36	74.68–99.36	93.63	80.56–99.60
NKp46	65.26	31.13–79.35	73.57	32.16 – 100
NKp30	30.24	10.24–52.63	34.77	24.32–98.49
2B4	98.67	34.6–99.91	99.84	37.78 – 100
DNAM	93.37	30.08–98.73	92.91	41.45–98.23

*IQ: interquartile*.

* *p < 0.05*.

Taken together, these results suggest that early EVL conversion from MMF is associated with an altered NK cell receptor balance, leading to an increase in the activation state and greater cytolytic activity of NK cells.

### Alterations in the immune phenotype arise soon after the introduction of EVL

**Figure 5:**
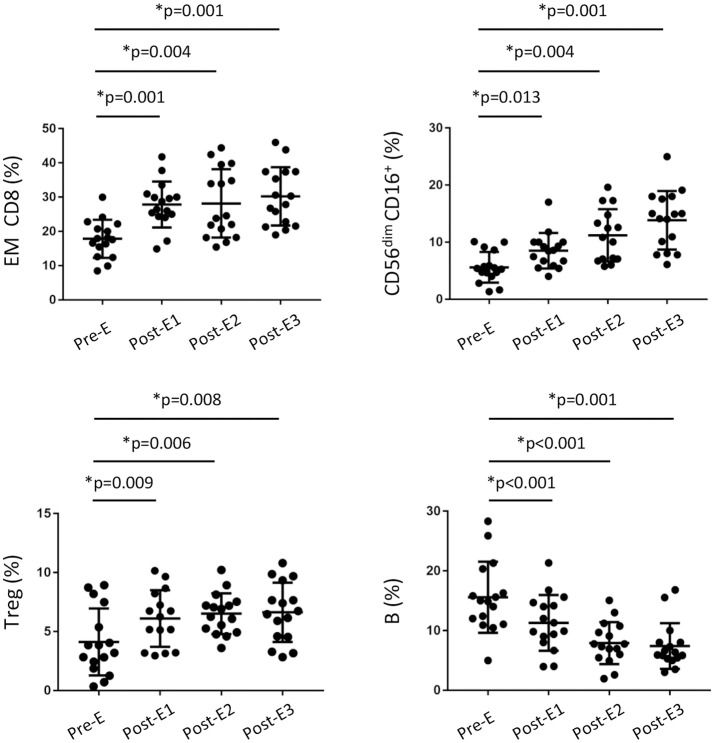
Changes in the immune phenotype of HT patients during the first year post-transplant from the prospective study. Distribution of CD8^+^ T, regulatory T, NK and B cells in peripheral blood from HT patients (*n* = 16) under EVL treatment from the third month post-transplant and follow-up at different times during the first year; before EVL initiation (Pre-E), and 1, 3, and 9 months after EVL conversion (Pre-E1, Pre-E2, Post-E3). Each circle represents one HT patient, and the mean and standard deviation are depicted as black bars. Differences between Pre-E and each time after EVL conversion (Post-E1, Post-E2 and Post-E3) were analyzed by the Wilcoxon test and *p < 0.05.

In light of our results, we wondered whether the increased frequency of cytotoxic CD8^+^ T and NK cells after early EVL conversion is a phenomenon observed only in the long term or if their effect is beginning immediately after EVL conversion. To address this, we prospectively analyzed a new cohort of HT patients (*n* = 16) who were converted early to EVL from MMF and from whom blood samples were obtained on several occasions during the first year post-HT: before EVL initiation (Pre-E) and at 1 (Post-E1), 3 (Post-E2), and 9 (Post-E3) months after EVL conversion. Full immunophenotyping, as previously described for the retrospective study, was carried out on each occasion. Results showed that the percentage of CD8^+^ T cells progressively increased during the first months after EVL conversion, but no changes were showed in the percentage of CD4^+^ T cells (Table S3). Moreover, in the CD8^+^ T cell compartment, naive CD8^+^ (N CD8^+^) T cells became less numerous in the months following the transplant but, by contrast, EM CD8^+^ T quickly enhanced after EVL conversion (Post-E1) and remained at levels similar to those observed in the retrospective study (Figure 5 and Table S3). Cytotoxic NK (CD56^dim^ CD16^+^) cells also became progressively more numerous during the first few months after EVL conversion, reaching levels at year (Post-E3) similar to those observed at long-terms (EVL-E group) (Figure 5 and Table S3). Thus, in accordance with the previous long-term results, the proportions of cytotoxic CD8^+^ T and NK cells are already increased soon after EVL conversion.

Surprisingly, we observed that the percentage of Treg cells had increased only one month after EVL conversion (Pre-E1), thereafter remaining stable throughout the rest of the study period (Figure 5 and Table S3). By contrast, the percentage of B cells gradually decreased during the first few months after transplant although no differences were detected between the EVL-E and MMF groups over the long term (Figure 5 and Table S3). These modifications early introduced after EVL initiation could condition the post-transplant immune response but further studies are needed to determine the functionality of those cells and their relevance.

### *IFNG* demethylation is associated with immune status after early EVL conversion

Alterations of the DNA methylation pattern of specific immune genes may condition the post-transplant immune response (33, 34). Thus, quantification of the DNA methylation level in key genes has been proposed as a biomarker for post-transplant follow-up, since it reveals changes at the molecular level that occur before phenotypic changes and clinical signs become apparent (35). We examined whether the percentages of cytotoxic CD8^+^ T and NK cells after early EVL conversion are associated with changes in the DNA methylation levels of cytotoxicity-related immune genes. To this end, we analyzed the DNA methylation levels of *IFNG (IFN-*γ*), FASLG* (Fas ligand), and *PRF1* (Perforin) genes in PBMCs isolated from patients of the prospective study at the same times (Pre-E, Post-E1, Post-E2, and Post-E3) that the immune phenotyping was carried out. PBMCs were used instead of purified CD8^+^ T and NK cells in order to find and easy and reliable biomarker that resembles the immunological status in peripheral blood observed by conventional techniques such as flow cytometry.

**Figure 6:**
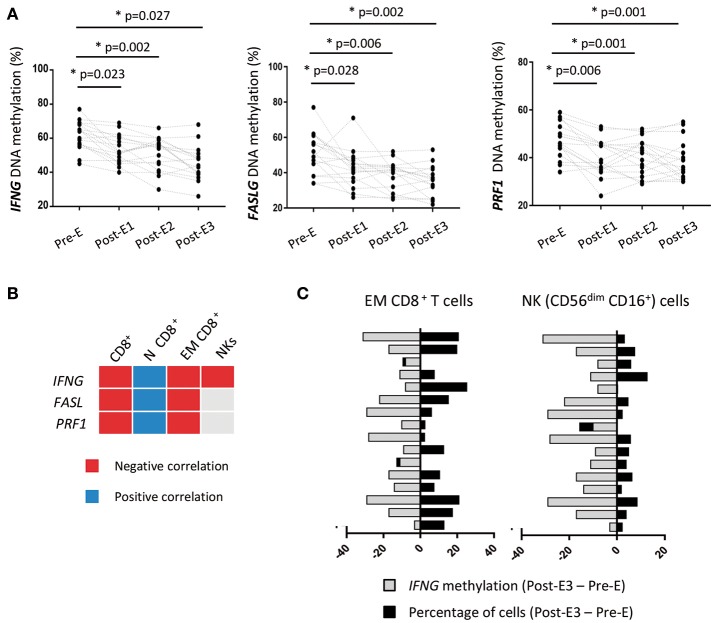
DNA methylation of immune genes in HT patients during the first year post-transplant from the prospective study. **(A)** The DNA methylation levels of *IFNG, FASLG*, and *PRF*1 genes were quantified by pyrosequencing in PBMCs isolated from HT patients (*n* = 16) under early EVL treatment and follow-up at different times during the first year; before EVL initiation (Pre-E), and 1, 3, and 9 months after EVL conversion (Pre-E1, Pre-E2, Post-E3). Each circle represents one HT patient and differences between Pre-E and each time after EVL conversion (Post-E1, Post-E2, and Post-E3) were analyzed by the Wilcoxon test. *p < 0.05. **(B)** Correlation between the DNA methylation level of each gene and the percentage of CD8^+^ T cell subsets and NK cells at all assayed times during the first year post-transplant. Negative and positive Pearson correlation coefficients indicate that demethylation of the gene is significantly associated with a higher or lower percentage of the cell subset, respectively. N, naive; EM, effector memory. **(C)** Association between the loss of *IFNG* DNA methylation and the increase in the percentage of EM CD8^+^ T and NK cells for each patient during the first year post-transplant (Post-E3–Pre-E times).

We observed that the *IFNG, FASLG*, and *PRF1* genes were significantly and gradually demethylated after early EVL conversion in all patients (Figure 6A). Moreover, demethylation of these genes was significantly correlated with an increased percentage of EM CD8^+^ T cells and a lower percentage of N CD8^+^ T cells (Figure 6B and Table S4). Demethylation of *IFNG* was the only gene found to be significantly associated with an increase in the percentage of cytotoxic NK cells (Figure 6B and Table S4). These results were seen in most patients (93.7% for NK and 87.5% for EM CD8^+^ T cells), suggesting that the *IFNG* DNA methylation level determined in PBMCs could be a useful biomarker for predicting the increase of cytotoxic cells induced after early EVL conversion (Figure 6C).

## Discussion

The efficacy of EVL-based therapies in HT patients has been clearly demonstrated in the last decade, showing their benefits mainly over the long term. Several randomized trials and observational studies have shown that the use of EVL in combination with low doses of CNI reduces CNI-induced nephrotoxicity, prevents CAV progression, and lowers the incidence of CMV infection. Some of these effects have been related to an expansion of Treg cells that could contribute to the development of a tolerant immune response, although the consequences of that remain unclear. However, their effects on other immune cells and, more importantly, their functional consequences are still poorly understood. In addition, the studies carried out to date consider very different EVL doses and administration strategies, which make it difficult to compare their results and to draw clear conclusions about the best immunosuppressive strategy.

In this study, we describe for the first time the phenotypic and functional changes in the immune response after EVL conversion from MMF while taking the timing of EVL initiation into account. Our findings show that early EVL initiation in HT patients: (*i*) supports the increase in the percentage of cytotoxic CD8^+^ T cells, allowing greater degranulation of cytotoxic molecules and higher number of INF-γ-producing cells under activation; (*ii)* reduces the expression of inhibitory receptors in NK cells, enhancing their activation state and lytic ability; (*iii)* raises the percentage of Treg cells only 1 month after EVL conversion, the levels being maintained over the long term. Furthermore, these effects on immune cells were not observed when EVL was initiated during maintenance periods, implying that the timing of EVL conversion may condition the immune response after HT and consequently the long-term outcome.

Cytotoxic CD8^+^ T and NK cells play a key role in long-term rejection and antibody-mediated rejection, respectively (36–38). In our study cohorts, we observed an increase of EM CD8^+^ T cells after long periods in patients receiving early EVL compared with those receiving MMF treatment. However, that significant enhancement was not detected when EVL was initiated later on (>1 year post-transplant), highlighting the importance of the timing of EVL initiation. Surprisingly, the increase in the proportion of this cell subset was detected only 1 month after early EVL initiation (Post-E1 time), and was correlated with fewer N CD8^+^ T cells. mTOR activity plays a key role in the transcriptional program that determines the fate of CD8^+^ T cells (23). Inhibition of mTOR promotes eomesodermin expression and induces the differentiation of memory CD8^+^ T cells with enhanced antigen-recall response. In fact, we showed that a higher number of CD8^+^ T cells from early EVL-treated patients have the capacity to express cytotoxic molecules (granzyme B, perforin, and IFN-γ) and liberate them into the extracellular space, as demonstrated by the high level of CD107a expression. The production of these molecules by EM CD8^+^ T cells had been previously associated with poorer survival of HT patients, and the expansion of EM CD8^+^ T cells by sirolimus in kidney transplantation patients showed a higher rejection rate (39, 40). Although EVL might be expected to inhibit the proliferation of both CD4^+^ and CD8^+^ T cells, our *in vitro* results showed this drug to be highly efficient at inhibiting the proliferation of CD4^+^ T cells. However, the blockage of CD8^+^ T cells is very limited, which might favor their expansion and differentiation. In fact, in our study no variations in the percentage of the CD4^+^ T cell subsets were observed.

We also observed an increase in the percentage of cytotoxic NK cells (CD56^dim^ CD16^+^) in HT patients after early EVL initiation, whereas other NK cell subsets were unchanged. Although few studies have analyzed the effect of EVL on NK cells, our results are consistent with those of a previous study showing that the lysis of renal tubular epithelial cells by CD8^+^ T and NK cells is efficiently inhibited by FK506 and prednisolone, but not by EVL (41). Other studies in kidney-transplanted patients reported an overexpression of NK cell lineage-specific transcripts and CD56^dim^ NK cells under sirolimus monotherapy compared with those treated only with cyclosporine or FK506, respectively (40, 42). To our knowledge, this is the first demonstration that early EVL conversion in HT patients is not efficient at inhibiting the expansion of cytotoxic NK cells, as shown by the progressive increase observed during the first few months following early EVL initiation. Under activation, these cells showed a greater ability to release cytotoxic molecules, as evinced by the CD107a expression. This could be due to the diminished expression of the inhibitory receptors KIR3DL1 and NKG2A on their cell surface. In this way, MPA (the active metabolite of MMF) has been proposed as the most potent inhibitor of NK cell cytotoxicity due to its capacity to increase the level of expression of the KIR inhibitory receptors whilst decreasing that of activating receptors (NKG2D, 2B4, NKp30, and NKp44) (43, 44). Thus, changes in the receptor repertoire induced early after EVL initiation could facilitate the activation of NK cells, thereby enhancing the NK-mediated immune responses. An open question is whether other activating receptors such as NKG2C, induced after CMV infection, could be modified by the immunosuppressive treatment and its relevance in the anti-CMV immune response. Eissens et al. (44) reported that NKG2C expression in NK cells does not change after *in vitro* culture for 5 days with different immunosuppressive drugs (Ciclosporine A, Rapamycin, and mycophenolic acid), but the effect of everolimus has not been evaluated.

Alterations in the DNA methylation patterns of immune genes are fundamental to the differentiation and function of immune cells, and their potential as biomarkers for identifying patients at high risk post-transplant is beginning to be understood (45–47). Loss of DNA methylation precedes an increase in the level of protein expression and thus in the cellular function. Thus, changes in the DNA methylation levels could be considered predictive biomarkers of clinical complications. In our study, we found a progressive demethylation of the *IFNG, FASLG*, and *PRF*1 genes during the first year after HT in patients with early EVL conversion. Our results are consistent with those from kidney-transplanted patients, in whom *IFNG* demethylation was associated with an increase of memory IFN-γ producing CD8^+^ T cells (48). Additionally, IFN-γ and perforin expression in endomyocardial biopsies have been proposed as a predictive marker of acute rejection in HT patients (49), and hypomethylation of *FASL* suggests an enrichment of cytolytic cells (33). In our cohort, a clear correlation between loss of methylation and an increased frequency of cytotoxic EM CD8^+^ T and NK (CD56^dim^ CD16^+^) cells was only reported for the *IFNG* gene in HT patients. Thus, we conclude that loss of *IFNG* methylation could facilitate expression of the gene and predict the increased cytotoxicity ability observed in HT patients after early EVL conversion. Moreover, its quantification by the easy, reliable, and highly stable pyrosequencing technique could be of used as a biomarker additional to flow cytometry.

One of the most widely reported immunological events after mTOR inhibitor treatment is the increase of Treg cells (50, 51). Accordingly, we observed an enhanced of Treg cells during the first month following EVL conversion, and over the longer term a significant difference from HT patients under MMF therapy was maintained. Unfortunately, we could not carry out functional assays with regulatory T cells to corroborate their functional ability to suppress activated T cells. Nonetheless, a recent study in liver transplantation shows that conversion from tacrolimus to everolimus or sirolimus not only increase the number of peripheral blood regulatory T cells but also preserve their suppressive capacity (52).

According to our results, it is too early to say whether these changes in the immune cells and their functionality directly affect transplant outcome. In our study, no clearly significant association was found between early EVL initiation and lesser amounts of ACR or CAV. However, EVL-E group patients exhibited stable renal function during the study period and a higher incidence of ACR was not detected, suggesting that early EVL initiation is a promising strategy to preserve renal function without compromising rejection. Moreover, a lower percentage of CMV infection was observed in these patients compared with those of the MMF treatment group. Previous results from our group showed that early EVL conversion combined with valganciclovir prophylaxis provides more effective protection from CMV infection than other treatments (53).

We are aware that our study is limited by the small number of patients considered. However, the results varied little among patients within the same group and the differences between groups were significant, which confers a degree of reliability on the results. Further studies in a larger cohort could confirm whether the limited efficacy of early EVL initiation to counteract the cytotoxic response mediated by memory CD8^+^ T and NK cells reported here might be useful for developing a potent viral immune response in those HT patients. Until then, the consequences of early EVL initiation after HT could be considered as a double-sided coin; on the one hand induces the Tregs expansion and reduces the B cell number that might contribute to a better graft outcome and, at the same time fails to block the cytolytic ability mediated by CD8^+^ T and NK cells.

We conclude that EVL conversion at 3 months post-HT is more efficient at inducing changes in the immune system that could improve the anti-CMV immune response, thereby ensuring a satisfactory outcome over the long term. Whether the limited efficiency for damaging the cytotoxic immune response of early EVL initiation strategies is associated with a better or worse allograft outcome, or a more robust specific anti-CMV immune response, needs to be clarified. In any case, early EVL conversion as a general strategy for all patients must be adopted with caution, since although it could be beneficial for patients with high-risk of CMV infection, it might be less efficient at suppressing the immune response to the graft in high-risk patients.

## Author contributions

BD-M was responsible for the recruitment and following of patients, analysis of clinical data, and collaborated to write the manuscript. PD-B and RC performed all flow cytometry staining experiments and analysis. MB performed the withdrawal of blood samples, patient care and quality control of clinical data. RR contributed to analysis including statistics and graphics and wrote the manuscript. VC-I performed all *in vitro* experiments and analysis. CM and JL were responsible for the transplantation and patient care including selection of patients. BS-A was responsible for the coordination of the study, quality control of experimental data, wrote the manuscript and is the corresponding author.

### Conflict of interest statement

The authors declare that the research was conducted in the absence of any commercial or financial relationships that could be construed as a potential conflict of interest.

## Footnotes

^1^Research Quality Association. *Good Clinical Practice (GCP) Regulations and Guidelines*. Available online at https://www.therqa.com/good-practices/good-clinical-practice/regulations-and-guidelines/. Update 2017.

^2^World Medical Association. Declaration of Helsinki. Available online at https://www.wma.net/policies-post/wma-declaration-of-helsinki-ethical-principles-for-medical-research-involving-human-subjects/.

## Supplementary material

Figure S1 Gating strategy used for peripheral blood immunophenotyping. Live peripheral blood mononuclear cells (PBMCs) were selected according to physical parameters (forward and side scatter) and by negative 7-AAD staining. **(A)** T lymphocytes were selected according to the CD3^+^ expression, then CD4^+^ and CD8^+^ T cells were identified. Further, combined expression of CCR7 (C-C motif chemokine receptor-7) and CD45RA surface markers was used to classify T cells in four subsets; naive (N: CD45RA^+^ CCR7^+^), central memory (CM, CD45RA^−^ CCR7^+^), effector memory (EM, CD45RA^−^ CCR7^−^) and terminally differentiated effector memory cells re-expressing CD45RA (TEMRA, CD45RA^+^ CCR7^−^). **(B)** NK cells subsets were identified first gating on living CD3^−^ T cells, and then considering the combined expression of CD56 and CD16 markers. **(C)** Regulatory T cells were characterized first gating living CD3^+^ and CD4^+^ T cells and further defined by the co-expression of CD25^high^ and CD127^low/−^.

Figure S2 Immune phenotype in peripheral blood of HT patients by treatment group. The absolute number (cells/μl) of the immune cell subsets was determined in 50 μl of peripheral blood obtained from HT patients at >1 year post-transplant who were receiving therapy with MMF (MMF group, *n* = 20) or who converted to EVL early (EVL-E, *n* = 12) or late (EVL-L, *n* = 8). Each circle represents one HT patient, and the mean and standard deviation are depicted as black bars. Significant differences between groups were determined by the Mann–Whitney *U*-test. ^*^*p* < 0.05.

Figure S3 Effect of EVL, FK506, and MMF on CD4^+^ and CD8^+^ T cell proliferation. **(A)** CD4^+^ and CD8^+^ T cells isolated from six healthy donors were stained with CFSE and activated with anti-CD3/CD28 antibodies in the presence of different amounts of EVL (1 and 10 μM), FK506 (100 nM and 1 μM) and MMF (100 nM and 1 μM) for5 days. Proliferation was quantified by gating CFSE-negative cells on CD4^+^ or CD8^+^ T lymphocytes. Results were normalized with respect to activated T cells and are shown as the mean and standard deviation of the six donors. Significant differences between CD4^+^ and CD8^+^ T cells were determined by Student's *t*-test orthe Mann–Whitney *U*-test. **(B)** CFSE division patterns of one representative donor after activation and proliferation inhibition with EVL, FK506, and MMF for 5 days. Numbers in the histogram indicate the percentage of dividing cells compared with controls (cells without activation). The lower panel shows the comparison of three drugs at 1 μM to inhibit the proliferation of CD4^+^ and CD8^+^ T lymphocytes.

Table S1 Baseline clinical characteristics of HT patients from the prospective study.

Table S2 Monoclonal antibodies used in flow cytometry.

Table S3 Immunophenotype of HT patients from prospective study at indicated times.

Table S4 Correlation between DNA methylation levels and cytotoxic immune cell subsets.

## Abbreviations

MMF: mycophenolate mofetil
EVL: everolimus
FK506: tacrolimus
CMV: cytomegalovirus
CAV: chronic allograft vasculopathy
mTOR: mammalian target of rapamycin
Tregs: regulatory T cells.
