# Supplementary figures and images for: Early Everolimus Initiation Fails to Counteract the Cytotoxic Response Mediated by CD8+ T and NK Cells in Heart Transplant Patients
Source: Front Immunol. 2018 Sep 26;9:2181. doi: 10.3389/fimmu.2018.02181 (PMC6168668; doi:10.3389/fimmu.2018.02181)

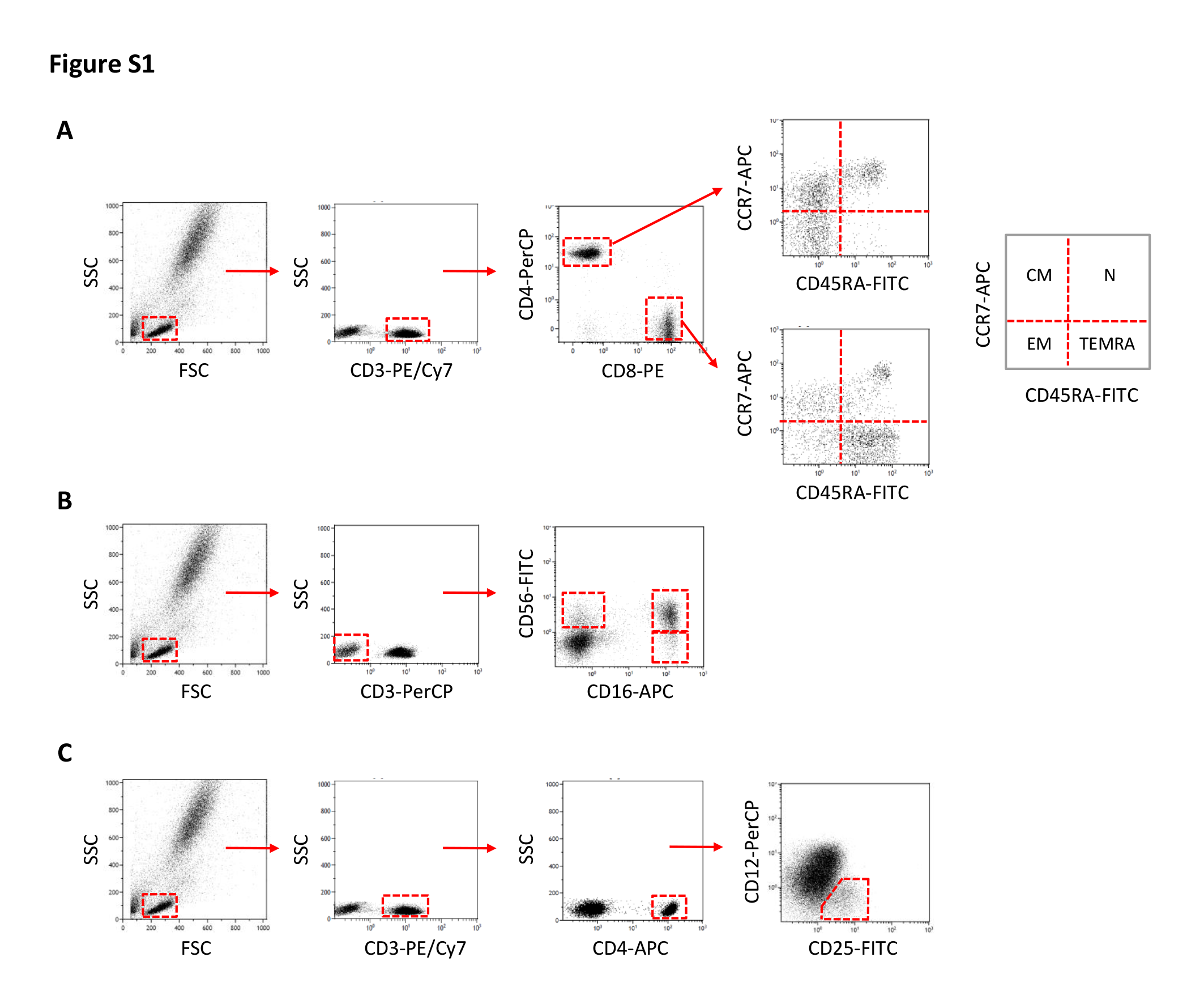

Supplement: Figure S1 — Gating strategy used for peripheral blood immunophenotyping. Live peripheral blood mononuclear cells (PBMCs) were selected according to physical parameters (forward and side scatter) and by negative 7-AAD staining. (A) T lymphocytes were selected according to the CD3+ expression, then CD4+ and CD8+ T cells were identified. Further, combined expression of CCR7 (C-C motif chemokine receptor-7) and CD45RA surface markers was used to classify T cells in four subsets; naive (N: CD45RA+ CCR7+), central memory (CM, CD45RA− CCR7+), effector memory (EM, CD45RA− CCR7−) and terminally differentiated effector memory cells re-expressing CD45RA (TEMRA, CD45RA+ CCR7−). (B) NK cells subsets were identified first gating on living CD3− T cells, and then considering the combined expression of CD56 and CD16 markers. (C) Regulatory T cells were characterized first gating living CD3+ and CD4+ T cells and further defined by the co-expression of CD25high and CD127low/−. [file Image_1.TIF]

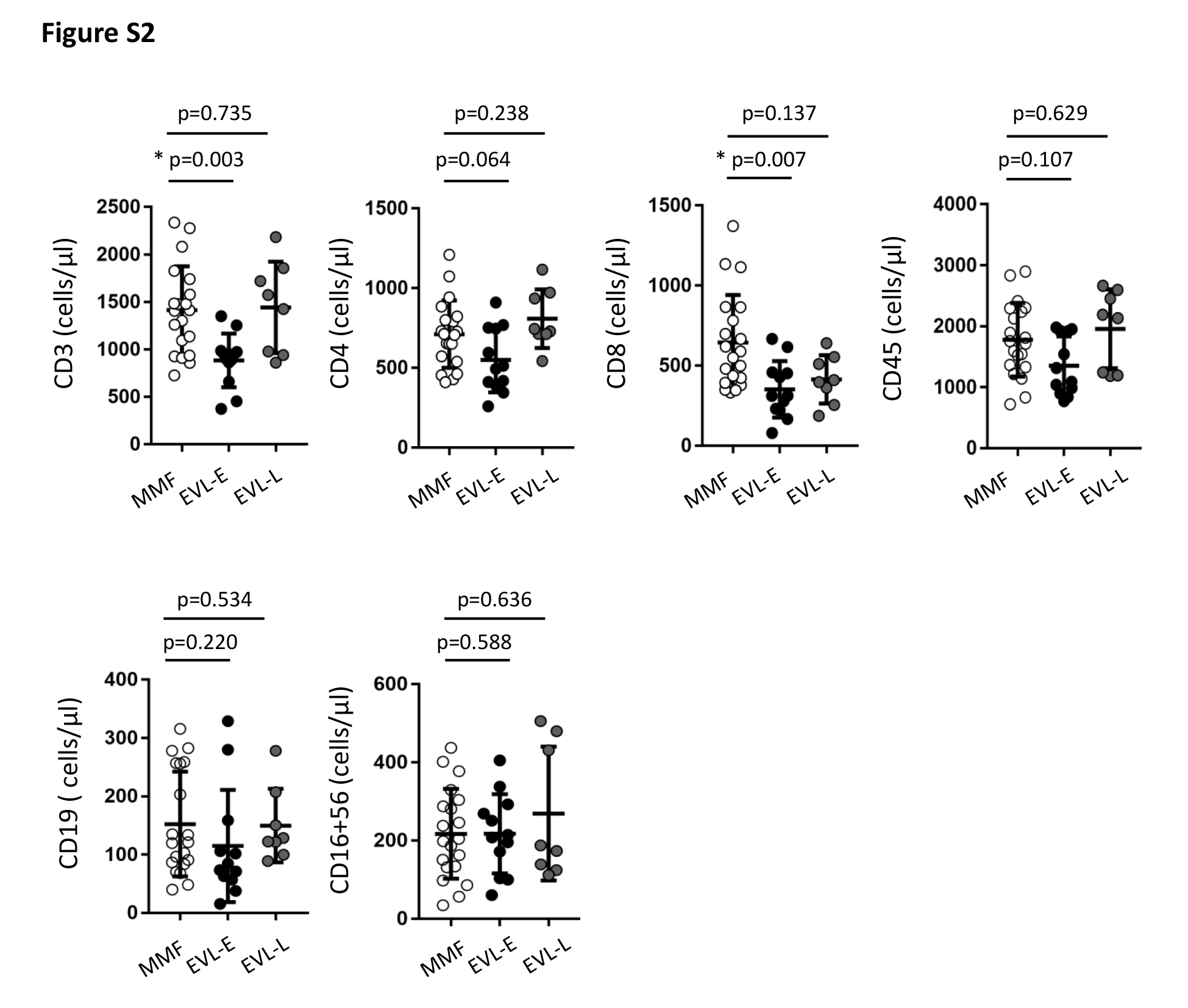

Supplement: Figure S2 — Immune phenotype in peripheral blood of HT patients by treatment group. The absolute number (cells/μl) of the immune cell subsets was determined in 50 μl of peripheral blood obtained from HT patients at >1 year post-transplant who were receiving therapy with MMF (MMF group, n = 20) or who converted to EVL early (EVL-E, n = 12) or late (EVL-L, n = 8). Each circle represents one HT patient, and the mean and standard deviation are depicted as black bars. Significant differences between groups were determined by the Mann–Whitney U-test. *p < 0.05. [file Image_2.TIF]

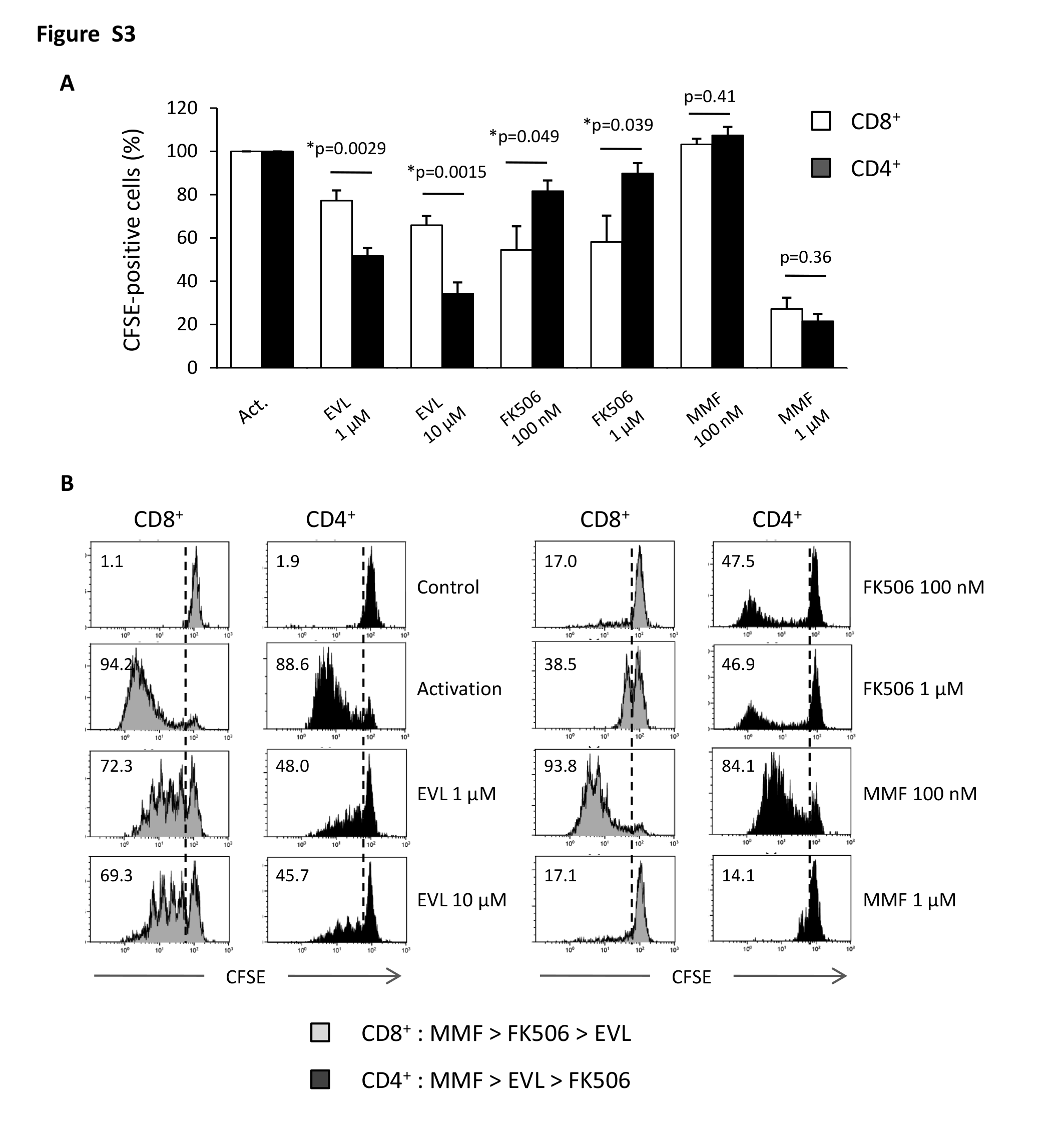

Supplement: Figure S3 — Effect of EVL, FK506, and MMF on CD4+ and CD8+ T cell proliferation. (A) CD4+ and CD8+ T cells isolated from six healthy donors were stained with CFSE and activated with anti-CD3/CD28 antibodies in the presence of different amounts of EVL (1 and 10 μM), FK506 (100 nM and 1 μM) and MMF (100 nM and 1 μM) for5 days. Proliferation was quantified by gating CFSE-negative cells on CD4+ or CD8+ T lymphocytes. Results were normalized with respect to activated T cells and are shown as the mean and standard deviation of the six donors. Significant differences between CD4+ and CD8+ T cells were determined by Student's t-test orthe Mann–Whitney U-test. (B) CFSE division patterns of one representative donor after activation and proliferation inhibition with EVL, FK506, and MMF for 5 days. Numbers in the histogram indicate the percentage of dividing cells compared with controls (cells without activation). The lower panel shows the comparison of three drugs at 1 μM to inhibit the proliferation of CD4+ and CD8+ T lymphocytes. [file Image_3.TIF]
