# Supplementary material for: Early Everolimus Initiation Fails to Counteract the Cytotoxic Response Mediated by CD8+ T and NK Cells in Heart Transplant Patients
Source: Front Immunol. 2018 Sep 26;9:2181. doi: 10.3389/fimmu.2018.02181 (PMC6168668; doi:10.3389/fimmu.2018.02181)
Supplement: Table S1 — Baseline clinical characteristics of HT patients from the prospective study. [file Table_1.DOCX]

**Table S1**.- Baseline clinical characteristics of HT patients from the prospective study.

|  | n = 16 |
| --- | --- |
| Donor age; median (IQ range), y | 49 (29-59) |
| Donor gender; n (M:F) | 9 : 7 |
| Recipient age; median (IQ range), y | 56 (44-69) |
| Recipient gender; n (M:F) | 12 : 4 |
| HLA mismatches; median (IQ range) | 5 (2-6) |
| Heart disease; n (%) |  |
| Dilated cardiomyopathy | 9 (56.25) |
| Ischemic cardiomyopathy | 5 (31.25) |
| Others | 2 (12.5) |
| Medical history; n (%) |  |
| Hypertension | 5 (31.25) |
| Diabetes | 1 (6.25) |
| Dyslipidemia | 4 (25) |
| Smoker | 1 (6.25) |
| Unknown | 5 (31.25) |
| Cellular rejection; n (%) | 5 (31.25) |
| Cytomegalovirus infection; n (%) | 1 (6.25) |
| Tumors; n (%) | 0 |
| Exitus; n (%) | 0 |

IQ: interquartile.
