# Supplementary material for: Early Everolimus Initiation Fails to Counteract the Cytotoxic Response Mediated by CD8+ T and NK Cells in Heart Transplant Patients
Source: Front Immunol. 2018 Sep 26;9:2181. doi: 10.3389/fimmu.2018.02181 (PMC6168668; doi:10.3389/fimmu.2018.02181)
Supplement: Table S2 — Monoclonal antibodies used in flow cytometry. [file Table_2.DOCX]

**Table S2.-** Monoclonal antibodies used in flow cytometry.

| Marker (mAb) | Clone | Company |
| --- | --- | --- |
| CD3-PerCP | SK7 | Biolegend |
| CD3-PerCP | UCHT1 | Biolegend |
| CD3-PE/Cy7 | OKT3 | Biolegend |
| CD4-APC | OKT4 | Biolegend |
| CD4-PerCP | OKT4 | Biolegend |
| CD8-PE | SK1 | Biolegend |
| CD16-APC | 3G8 | Biolegend |
| CD25-FITC | BC96 | Biolegend |
| CD28-FITC | CD28.2 | Biolegend |
| CD45RA-FITC | HI100 | Biolegend |
| CD56-FITC | MEM188 | Biolegend |
| CD127-PerCP | A019D5 | Biolegend |
| CD158a-PE | HP-MA4 | Biolegend |
| CD158b-PE | DC27 | Biolegend |
| CCR7-APC | G043h7 | Biolegend |
| DNAM-PE | 11A8 | Biolegend |
| FoxP3-PE | 259D | Biolegend |
| KIR2DL1-FITC | DX9 | Biolegend |
| NKG2A-FITC | REA110 | Myltenyi Biotech |
| NKG2D-PE | 1D11 | Biolegend |
| NKp30-PE | P30-15 | Biolegend |
| NKp46-FITC | 9E2 | Biolegend |
| 2B4-FITC | C1.7 | Biolegend |
