# Supplementary material for: Early Everolimus Initiation Fails to Counteract the Cytotoxic Response Mediated by CD8+ T and NK Cells in Heart Transplant Patients
Source: Front Immunol. 2018 Sep 26;9:2181. doi: 10.3389/fimmu.2018.02181 (PMC6168668; doi:10.3389/fimmu.2018.02181)
Supplement: Table S3 — Immunophenotype of HT patients from prospective study at indicated times. [file Table_3.DOCX]

|  | **Pre-E** | | **Post-E1** | | **Post-E2** | | **Post-E3** | |  |
| --- | --- | --- | --- | --- | --- | --- | --- | --- | --- |
| CD45; cells/µl | 1081.88 (412.36-2571.21) | | 1736.78 (757.15-3158.64) | | 1104.85 (772.22-2364.61) | | 1125.88 (592.09-1750.06) | |  |
| CD3; cells/µl | 775.73 (349.4-2136.41) | | 1154 (609.57-2706.29) | | 1027.03 (586.79-2035.07) | | 1014.24 (373.07-1570) | |  |
| CD4 ; cells/µl | 466.84 (208.46-1171.67) | | 648.44 (356-1972.11) | | 588.13 (395.29-1418.07) | | 555.57 (258.777-910) | |  |
| CD8 ; cells/µl | 317.41(112.19-782.38) | | 488.74 (169.2-1000.38) | | 374.69 (139.13-856.97) ^*^ | | 414.5 (80.47-928) | |  |
| NKs ; cells/µl | | 40.88 (13.58-144.54) | | 142.2 (44-296)^*^ | | 149.78 (80.26-310.61) ^*^ | | 189.15 (42-369.33) ^*^ | |
| B ; cells/µl | 170.09 (11.82-322.04) | | 177.72 (30.04-279.37) | | 106.96 (15.57-236.25) ^*^ | | 79.31 (7.09-285.63) ^*^ | |  |
| CD3; % | 81.52 (65.55-87.84) | | 80.22 (68-85.88) | | 84.38 (65.36-89.17) ^*^ | | 75.34 (62.93-88.07) | |  |
| CD4; % | 49.01 (27.02-70.82) | | 44.36 (34.09-69.55) | | 45.55 (30.87-70.98) | | 43.67 (29-70.24) | |  |
| N CD4; % | 42.42 (17-72.37) | | 46.59 (16.62-69.99) | | 40.72 (13.37-62.58) | | 37.81 (15.56-69.24) | |  |
| CM CD4 ; % | 27.87 (4.96-40.24) | | 23.09 (4.79-58.73) | | 33.44 (5.15-51.25) | | 31.56 (11.05-54.17) | |  |
| EM CD4; % | 23.01 (8.81-48.81) | | 22.42 (10.28-41.03) | | 21.45 (4.38-60.88) | | 26.16 (2.13-60.94) | |  |
| TEMRA CD4; % | 1.76 (0.37-30.12) | | 1.95 (0.63-23.89) | | 3.10 (0.11-17.24) | | 2.7 (0.15-21.93) | |  |
| CD8; % | 23.11 (10.39-40.93) | | 28.18 (10.77-43.45) ^*^ | | 28.90 (12.01-48.48) ^*^ | | 34.17 (13.19-54.39) ^*^ | |  |
| N CD8; % | 35.80 (10.39-66.71) | | 27.73 (7.07-71.01) | | 16.37 (6.07-53.08) ^*^ | | 10.70 (5.28-68.13) ^*^ | |  |
| CM CD8; % | 7.2 (0.94-21.56) | | 7.27 (0.45-23.13) | | 8.6 (0.48-31.51) | | 6.2 (1.81-29.99) | |  |
| EM CD8; % | 17.77 (8.47-29.98) | | 27.57 (14.89-41.79) ^*^ | | 24.18 (15.42-44.41) ^*^ | | 29.09 (18.99-45.97) ^*^ | |  |
| TEMRA CD8; % | 35.16 (7.21-72.78) | | 36.08 (2.62-64.32) | | 42.92 (12.16-77.21) | | 42.2 (3.87-69.61) | |  |
| NKs (CD56^dim^ CD16); % | 4.99 (1.34-10) | | 7.95 (4-17) ^*^ | | 10.44 (5.75-19.61) ^*^ | | 14.55 (6.10-24.99) ^*^ | |  |
| B; % | 14.43 (4.98-28.32) | | 9.65 (3.97-21.33) ^*^ | | 7.76 (1.93-15.05) ^*^ | | 5.84 (0.10-16.81) ^*^ | |  |
| Treg ; % | 3.84 (0.30-8.93) | | 6.49 (2.96-10.16) ^*^ | | 6.92 (3.60-10.22) ^*^ | | 6.74 (3.17-10.81) ^*^ | |  |

**Table S3.-**Immunophenotype of HT patients from prospective study at indicated times.

^*^ p<0.05 compared to Pre-E time ; median (interquartile range).
