# Supplementary material for: Early Everolimus Initiation Fails to Counteract the Cytotoxic Response Mediated by CD8+ T and NK Cells in Heart Transplant Patients
Source: Front Immunol. 2018 Sep 26;9:2181. doi: 10.3389/fimmu.2018.02181 (PMC6168668; doi:10.3389/fimmu.2018.02181)
Supplement: Table S4 — Correlation between DNA methylation levels and cytotoxic immune cell subsets. [file Table_4.DOCX]

**Table S4.-** Correlation between DNA methylation levels and cytotoxic immune cell subsets.

|  |  | **CD8^+^** | **N CD8^+^** | **EM CD8^+^** | **NK** |  |
| --- | --- | --- | --- | --- | --- | --- |
| ***IFNG*** | Pearson correlation | -0.813 | 0.761 | -0.424 | -0.476 | |
|  | *p* | ≈***0.000*** | ≈***0.000*** | ***0.004*** | ***0.004*** | |
| ***PRF1*** | Pearson correlation | -0.371 | 0.279 | -0.388 | -0.255 | |
|  | *p* | ***0.004*** | ***0.036*** | ***0.002*** | *0.113* | |
| ***FASL*** | Pearson correlation | -0.669 | 0.671 | -0.398 | -0.191 | |
|  | *p* | ≈***0.000*** | ≈***0.000*** | ***0.002*** | *0.231* | |
